# Alpha-band echoes evoked by contrast and luminance changes emerge in and travel out from early visual cortex

**DOI:** 10.1167/jov.26.1.16

**Published:** 2026-01-28

**Authors:** Audrey Morrow, Elise Turkovich, Soorya Sankaran, April Pilipenko, Jason Samaha

**Affiliations:** 1 Psychology Department, University of California, Santa Cruz, Santa Cruz, CA, USA; 2 Department of Psychiatry and Behavioral Sciences, University of California, San Francisco, San Francisco, CA, USA

**Keywords:** traveling waves, alpha oscillations, cross-correlation, contrast, luminance

## Abstract

How stimulus properties are processed in the human brain over time is critical to how we engage in dynamic everyday environments. To understand how changes in basic stimulus properties relate to changes in human electrical brain activity over time, previous work has estimated the brain's temporal response function (TRF) by cross-correlating random luminance sequences with electroencephalogram (EEG) signals at various lags to approximate the brain's response to temporal changes in luminance. Using this technique, it was found that luminance changes produce long-lasting “echoes” in the alpha frequency range. However, the neural origin of these echoes and the precise stimulus features that induce them have not been extensively studied. We measured TRFs in response to luminance and contrast changes. Additionally, the fact that EEG responses generated in the primary visual cortex (V1) have a unique pattern of polarity reversal depending on the visual field location (with upper stimuli projecting negatively and lower projecting positively) allowed us to test whether the TRFs generated from upper or lower visual field stimulation were counter-phased, as would be expected if the echoes were generated within V1. We found a luminance echo lasting ∼1 s in the alpha frequency and contrast echoes lasting only around 300 ms. For both stimuli, the TRF was initially counter-phased between upper and lower visual fields but quickly became in phase after ∼100 ms. Our findings demonstrate the existence of contrast (in addition to luminance) echoes in the alpha band, which appear to emerge from V1, perhaps as a traveling wave.

## Introduction

Alpha-band (7–14 Hz) activity is prominent over the visual cortex and is associated with aspects of visual processing, from the temporal resolution of visual perception (Battaglini et al., 2020; Haarlem, Mitchell, Jackson, & O'Connell, 2024; Samaha & Postle, 2015) to shifts in spatial attention (Kelly, Gomez-Ramirez, & Foxe, 2009; Thut, Nietzel, Brandt, & Pascual-Leone, 2006) and even the likelihood of perceiving visual stimuli (Busch, Dubois, & VanRullen, 2009; Mathewson, Gratton, Fabiani, Beck, & Ro, 2009; Pilipenko & Samaha, 2024). However, questions remain about how those oscillations may facilitate the maintenance and flow of visual information through the visual hierarchy. Prior research has estimated the brain's temporal response function (TRF) to luminance changes and found a long-lasting “echo” in the alpha-band range, potentially revealing a mechanism for retention and integration of luminance information (VanRullen & Macdonald, 2012). In their study, participants observed a stimulus that varied randomly in luminance values from black to white (on a black background). The luminance values from each trial were then cross-correlated with the corresponding electroencephalogram (EEG) signal at various lags to estimate the TRF. The result showed a clear oscillating TRF whereby the correlation values oscillated between positive and negative values across lags at a frequency that correlated with each participant's individual alpha frequency (IAF). Further experiments showed that the amplitude of these luminance echoes increased with attention (VanRullen & Macdonald, 2012) and with sequence predictability (Chang, Schwartzman, VanRullen, Kanai, & Seth, 2017), suggesting that alpha echoes may play a role in briefly maintaining relevant visual information for later cognitive and perceptual processing (Chang et al., 2017; VanRullen & Macdonald, 2012).

These alpha-band echoes seem to be unique to visual processing, with no apparent echo in audition (İlhan & VanRullen, 2012) and a beta-band echo in somatosensation (Chota, VanRullen, & Gulbinaite, 2023). However, the specific visual features that induce visual echoes are not known. Prior work has focused on TRFs derived from luminance changes using a grayscale stimulus on a black background. However, such stimuli also vary in contrast as the stimulus becomes lighter relative to the background. Prior work on luminance echoes did not isolate luminance from contrast changes, making it unclear whether these alpha echoes emerge from luminance changes, contrast changes, or both. While it is true that prior studies have used contrast modulations to study TRFs using a method termed “VESPA,” the VESPA method has notable differences from the approach taken here to analyze alpha-band echoes. The VESPA method has focused on characterizing the first few peaks in the visual evoked response, rather than the longer-lasting echo. As such, the VESPA approach typically uses short sliding windows of about 500 ms to estimate the TRF (Lalor, Kelly, & Foxe, 2012), which would exclude much of the late echo component. While previous findings using VESPA provide insight into the brain's TRF to contrast modulation, reflecting early visual components, the method has not been used to test whether a longer alpha-band TRF occurs in response to contrast versus luminance stimuli. Thus, the first aim of this study was to evaluate whether alpha echoes emerged from the processing of a single stimulus feature, contrast. If alpha echoes are reflective of the maintenance and transference of visual information, there should be some emergence of an alpha echo for contrast changes, although the echo itself may be less prominent or long-lasting compared to an echo reflecting two visual features, contrast and luminance.

In order for these alpha echoes to support further perceptual processing, it is important that they not only be maintained for long periods of time in visual areas but also be spread to other areas. Recent research has supported the notion that alpha echoes travel by demonstrating that the phase of the alpha echo varied across midline electrodes in a manner suggestive of a wave traveling from posterior to anterior regions (Lozano-Soldevilla & VanRullen, 2019). Despite this evidence of alpha echoes as a traveling wave across the cortex, the neural generators have been difficult to infer from EEG. Specifically, it has been argued that the phase differences between posterior and more anterior electrodes, as observed in Lozano-Soldevilla and VanRullen (2019), are not due to a genuine traveling wave phenomenon across cortex but rather are the result of two separate posterior oscillatory generators, one at an occipital location and another at a parietal location, whose signals mix in a weighted manner across the anterior-posterior axis (Zhigalov & Jensen, 2023). Thus, the second aim of this study was to further investigate the traveling nature of the echoes by seeking additional sources of evidence beyond topographical phase shifts.

**Figure 1.**
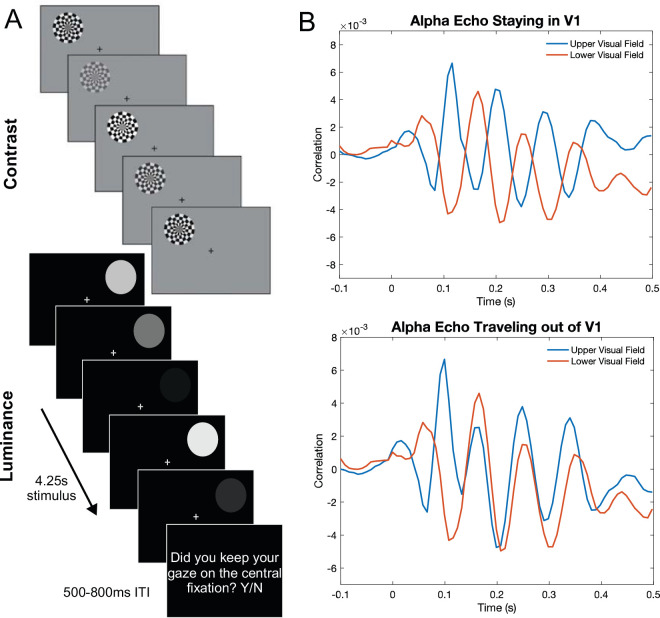
(**A**) Task diagrams showing an example of the contrast and luminance stimuli updating with each screen refresh. Stimuli changed contrast or luminance values at 120 Hz and were presented for 4.25 s, followed by a question asking whether participants maintained their gaze on the central fixation. (**B**) A schematic of theoretical alpha echoes for upper (blue) and lower (red) visual field stimuli if the echoes remained in primary visual cortex (V1; top) versus if they were generated in, but then traveled out of, V1 (bottom).

Here, we used stimulus locations known to activate the upper and lower banks of the calcarine sulcus in V1, which thereby evokes a distinct polarity reversal of surface potentials (Kelly, Vanegas, Schroeder, & Lalor, 2013). Activation of visual areas V2 and V3 evokes polarity reversals that seem to be contralateral for upper visual field (UVF) stimuli in measured data or positive for upper and negative for lower visual field (LVF) stimuli in simulated data (Ales, Yates, & Norcia, 2013; Ales, Yates, & Norcia, 2010). However, these patterns vary from V1 polarity reversals, which reflect negative activation for upper and positive activation for LVF stimuli, making the activation of V1 uniquely identifiable (Kelly et al., 2013). Specifically, this unique activation is reflected in the C1 event-related potential (ERP), which peaks between 60 and 90 ms and is thought to characterize the earliest afferent sweep of visual information in V1 (Clark, Fan, & Hillyard, 1994), consistent with dipole source localization of the C1 (Di Russo, Martínez, & Hillyard, 2003; Di Russo, Martínez, Sereno, Pitzalis, & Hillyard, 2002). Importantly, the polarity is reversed for upper and lower visual field stimuli such that UVF stimuli evoke a negative-going response, and LVF stimuli evoke a positive-going response (Ales, Carney, & Klein, 2010; Di Russo et al., 2003; Mohr, Geuzebroek, & Kelly, 2024). This specific pattern of polarity reversal is expected on the basis of the arrangement of neurons along the folds of the calcarine sulcus in V1 but, importantly, not extrastriate areas (Kelly et al., 2013). Therefore, if alpha echoes are both generated and confined within V1, we would expect to see the initial C1 polarity reversal maintained when comparing upper and lower visual field (VF) echoes, but if the alpha echo is indeed traveling out of V1, that polarity reversal should only last for the duration of the early sensory response at stimulus onset before the echoes become phase-synchronized (Figure 1).

## Method

### Procedure

Two tasks were completed across 2 days of EEG testing, both approved by the University of California, Santa Cruz (UCSC) Institutional Review Board. Eleven participants (*M*_age_ = 25.27, *SD*_age_ = 5.78) familiar with visual psychophysical tasks were recruited from the Samaha Lab at UCSC for the contrast version of the task, and 10 of those participants (*M*_age_ = 26.50, *SD*_age_ = 6.06) also performed the luminance version of the task. Based on a short demographic questionnaire, all participants reported normal or corrected-to-normal vision and identified as the following: 45.45% female, 36.36% male, 18.18% nonbinary, and 45.45% Caucasian, 18.18% Asian, 18.18% Persian, 9.09% Indian, and 9.09% Filipino. During the tasks, participants were asked to maintain their eyes on a central fixation while attending to a stimulus that appeared on the screen in one of the four visual quadrants. The only task was for participants to report, at the end of each trial, whether they maintained central fixation throughout the stimulus presentation using a button press (“Y” for yes and “N” for no). Finally, 2 min of EEG data were recorded at the end of the task while participants sat still with their eyes closed in order to get a clear measure of IAF for each participant.

### Task design

The luminance and contrast tasks were similar in design. Both tasks consisted of 600 trials of a 4.25-s stimulus presentation on each trial. Stimuli were presented on a gamma-corrected VIEWPixx EEG monitor (1,920 × 1,080 resolution, 120 Hz refresh rate) and generated using PsychToolbox 3 (Brainard, 1997). The fixation was a central crosshair made up of two lines that were 0.5 degrees of visual angle (DVA) in length. UVF stimuli were presented 3 DVA above fixation and 8 DVA to the left or right of fixation (eccentricity: 8.54°; polar angle: 159.4°/20.5°). LVF stimuli were presented 5 DVA below fixation and 7 DVA to the left or right of fixation (eccentricity: 8.6°; polar angle: 144.4°/35.5°). These stimulus locations were selected based on the visual field locations that have been shown to elicit the strongest C1 ERP in our previous work (Morrow, Pilipenko, Turkovich, Sankaran, & Samaha, 2024), as well as that of others (Clark et al., 1994), thus maximizing V1 contributions to the EEG response. All stimuli were shown in circular apertures of 6 DVA in diameter.

The luminance task closely followed the original study by VanRullen and Macdonald (2012) and presented white circles, varying in luminance, on a black background. The contrast task used annular checkerboard stimuli that were composed of 8 circles and 24 radial lines that varied in contrast on a gray background. For each trial, a random luminance or contrast sequence was generated and Fourier transformed. In the Fourier domain, the energy at each frequency was equated and inverse Fourier transformed to generate a unique random temporal sequence with equal energy at all frequencies on every trial. These sequences were used to update the luminance or contrast value of the stimulus on every refresh of the monitor (120 Hz).

### Data processing

For both tasks, the EEG was sampled and processed in the same way. The EEG was recorded using a 64-channel actiCHamp EEG system at a sampling rate of 1,000 Hz using “FCz” as the online reference electrode. Data were processed in MATLAB using the EEGLAB toolbox (Delorme & Makeig, 2004). We high-pass filtered the data using a Hamming window-sinc FIR filter with a low cutoff of 0.1 Hz. The data were downsampled to 120 Hz to match the screen refresh rate and then median re-referenced. Trials were epoched from −1 to 5 s relative to the target presentation to capture the entire 4.25-s stimulus and about 1 s of pre- and poststimulus data. Manual inspection of data was performed to remove noisy trials and trials where eye blinks overlapped the target presentation (number of trials removed luminance: *M* = 81.20, *SEM* = 58.57; contrast: *M* = 71.27, *SEM* = 54.82). Electrodes were interpolated due to excessive high-frequency noise that would have independently resulted in the rejection of more than 10% of trials (luminance: *M* = 2.90, *SEM* = 2.03; contrast: *M* = 3.27, *SEM* = 1.49). The INFOMAX algorithm (EEGLAB function binica.m) was then used to first perform a principal components analysis to correct the rank of the data and then perform an independent components analysis to remove ocular artifacts. Data were baseline corrected using a 500-ms pretarget window such that the mean voltage from the pretarget window was subtracted from the whole trial data, following standard EEG preprocessing steps. The resting state data were processed using all of the same steps, except that the ongoing 2-min recording was epoched into 1-s trials, no ICA was performed on the eyes-closed data, and no baseline correction was applied.

### Data analysis

**Figure 2.**
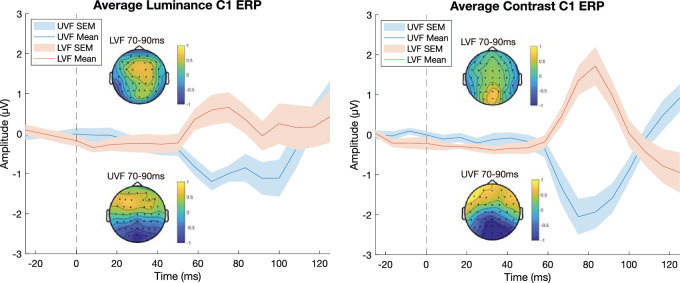
Plots of the average C1 ERP in response to stimulus onset for each stimulus feature (left: luminance, right: contrast) using each participant's best C1 electrode. Topoplots capturing the mean C1 ERP over 70–90 ms are shown for each stimulus and visual field (top: LVF; bottom: UVF). A clear polarity reversal is seen in both stimulus conditions as a function of the visual field location of the stimulus, which, along with the early timing of the response, suggests striate genesis.

As a sanity check of whether our choice of stimulus locations reliably elicited C1 responses with reversed polarity, we computed the ERP time-locked to the onset of each stimulus sequence (see Figure 2). For each participant, we examined the electrode with the strongest C1 response (luminance: 100% POz; contrast: 64% POz, 27% Pz, 9% P2) and broke these responses down by stimulus feature (contrast or luminance) and by visual field location (UVF/LVF). It should be noted that, while it is common to observe the C1 at electrode Oz, the topography can vary widely across individuals (Kelly, Gomez-Ramirez, & Foxe, 2008) and, depending on stimulus location, can often be strongest at POz (Iemi et al., 2019), as was the case in our data. Additionally, we strove to select the electrode that had the best response for *both* UVF and LVF stimuli, meaning it was not always the best electrode for the UVF or LVF C1 response when considering them independently.

To then compute the TRF, we first *z*-scored the contrast and luminance sequences from 0.50 to 3.25 s and the EEG data from 0.30s to 4.30 s poststimulus on each trial. The EEG data window was longer to account for edge artifacts. Notably, we did not include stimulus onset and offset responses in the cross-correlation in order to capture the echo generated by the steady-state response to the stimulus. We then cross-correlated the EEG data and stimulus sequence on each trial at 180 time point lags from −0.1 to +1 s. For the TRF analysis, we used the same electrode that was identified for each subject as having the clearest C1 response to stimulus onset.

To statistically evaluate the presence and duration of the echoes, we used a permutation approach where, for each subject, we shuffled the trial mapping between stimulus sequences and EEG data. We computed 2,000 permutations of the shuffled trial data per participant to compare against the true, trial-aligned TRFs. The amplitude of real and permuted alpha echoes was extracted in the same way for contrast and luminance stimulus sequences. We then filtered the echo data separately for UVF and LVF using a Hamming-windowed sinc FIR filter at a 120 Hz sampling rate (the screen refresh rate) and centered on participants’ IAF ± 2 Hz (derived from a fast fourier tansform (FFT) of resting data). Then, a Hilbert transform was applied to the filtered UVF and LVF echoes for both the real and permuted data. Using the Hilbert-transformed data, we calculated the amplitudes of each echo by taking the absolute value at each time point. To evaluate the time points at which the real echoes were statistically significant, we compared the real echo amplitude to the 95th percentile of the permuted echo amplitudes and used cluster size correction to control for multiple comparisons. Specifically, we evaluated whether each permuted echo's amplitude was greater than the 95th percentile of the permuted echo amplitudes from −100 to 1,000 ms relative to lag 0. We then saved the largest significant temporal sizes from each permutation to be used as a threshold for multiple comparisons correction. Only clusters in the real data that exceeded the 95th percentile of the distribution of cluster sizes in the permuted data were considered statistically significant. We used the same approach as outlined above to also compare amplitude differences between the upper and lower VF echoes for each task. After computing the amplitudes, we took the difference of upper and lower VF echo amplitudes for each stimulus type (contrast and luminance) for real and permuted echoes, then computed clusters and retained those above the 95th percentile of significant permuted clusters.

To evaluate the phase differences of the echoes across time, we used a bootstrap sampling method where we selected 5,000 random samples of 10 participants with replacement. For each sample, we took the mean of upper and lower VF echoes, filtered the data using a Hamming-windowed sinc FIR filter centered on the bootstrap sample's mean peak alpha frequency ± 2 Hz, then performed a Hilbert transform. Using the CircStats toolbox (Berens, 2009), we computed the circular distance between the upper and lower VF echoes to get phase vectors at each time point and bootstrap that captured the phase differences between the echoes. We then compared the bootstrapped phase differences to the actual echoes’ average phase differences from a −100- to 0-ms baseline window using a two-tailed approach, assuming that the echo could be more in phase or out of phase than the baseline. We computed *p*-values as the nonparametric probability of observing the bootstrapped phase difference at each time point relative to the mean across the baseline. Note that a bootstrap approach was taken for the analysis of phase values instead of a permutation approach since the null hypothesis of no systematic phase difference is captured by a uniform distribution around the unit circle rather than one condition being larger or smaller than another, as was the case with previous hypotheses we tested with permutations. The bootstrapping approach thus allowed us to compare the variability in observed phase differences to an empirical null value derived from the baseline window.

To further extend the comparison of upper and lower VF echoes, we also analyzed the dominant alpha frequency of the first 500 ms of each echo. The echoes were zero-padded and tapered with a Hamming window and then linearly detrended. We then performed an FFT to extract power at each channel for each trial separately for upper and lower VF echoes. Finally, we used each participant's best C1 electrode to search for their peak echo frequency within the alpha-band range of 7–14 Hz. The dominant alpha-band frequency from each visual field echo was compared using a Spearman correlation for each task. As an exploratory measure, we also compared the dominant frequencies of the upper and lower VF echoes for contrast and luminance stimuli to IAF extracted from the eyes-closed resting state data using a Spearman correlation. We also did this for the average dominant frequency of each echo, collapsing across visual fields.

## Results

**Figure 3.**
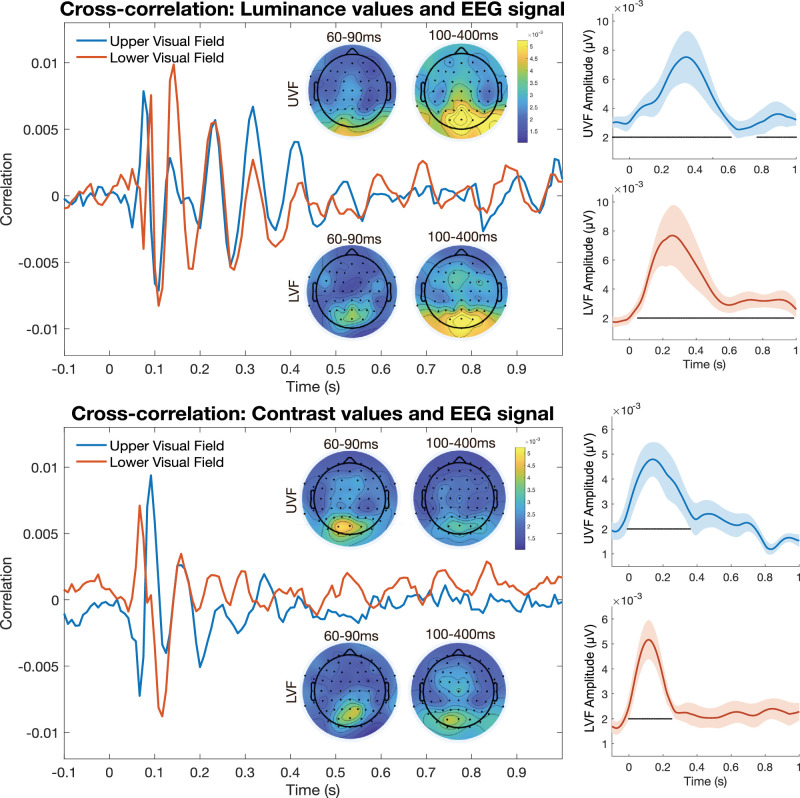
Average alpha-band echoes over time for UVF (blue lines) and LVF (red lines) stimuli following changes in luminance (top) and contrast (bottom). Plots showing the topography of each echo's alpha amplitude for early (60–90 ms) and late (100–400 ms) intervals are shown for the different stimulus locations (upper: UVF; lower: LVF). Average amplitudes for the UVF and LVF echoes are shown on the right side of the figure, with points indicating significant amplitudes after cluster correction. Shaded regions indicate the standard error of the mean (*SEM*). Note that the group-averaged echoes appear shorter than the amplitude time series due to some phase cancellation caused by averaging over subjects.

We found a clear cross-correlation in the alpha frequency range between the EEG signal and unit changes in luminance and contrast (see Figure 3). Alpha echoes for the UVF and LVF luminance stimuli lasted roughly 1 s, and echoes for the UVF and LVF contrast stimuli lasted roughly 0.3 s. To assess whether these echoes were generated by the random luminance or contrast sequences, as opposed to by chance or due to an autocorrelation in the EEG signal, we statistically evaluated the echo amplitudes. Amplitudes for the luminance echoes were significantly greater than the permuted luminance echoes (*p* < 0.05, cluster-corrected) between the time points −0.100–0.592 s and 0.750–1.033 s for the UVF stimuli and 0.050–0.958 s for the LVF stimuli (Figure 3, top right panels). Amplitudes for the contrast echoes were significantly greater than the permuted contrast echoes (*p* < 0.05, cluster-corrected) between time points 0–0.358 s for the UVF stimuli and 0–0.250 s for the LVF stimuli (Figure 3, bottom right panels). Note that the group-averaged alpha echo may appear shorter in duration than the amplitude analysis suggests, but keep in mind that any phase differences in the TRF across time will diminish the amplitude of the group-averaged echo, and thus the amplitude analysis shown in the rightmost panels in Figure 3 (performed on individual subjects prior to averaging) is a better representation of the echo duration. Notably, the upper and lower visual field stimuli produced echoes with very comparable amplitudes and time courses for both the contrast and luminance stimuli. Indeed, the upper and lower visual field echoes were only significantly different between 0.975 and 1.258 s in response to contrast stimuli (*p* < 0.05, cluster-corrected) and were not significantly different at any time point for luminance stimuli.

**Figure 4.**
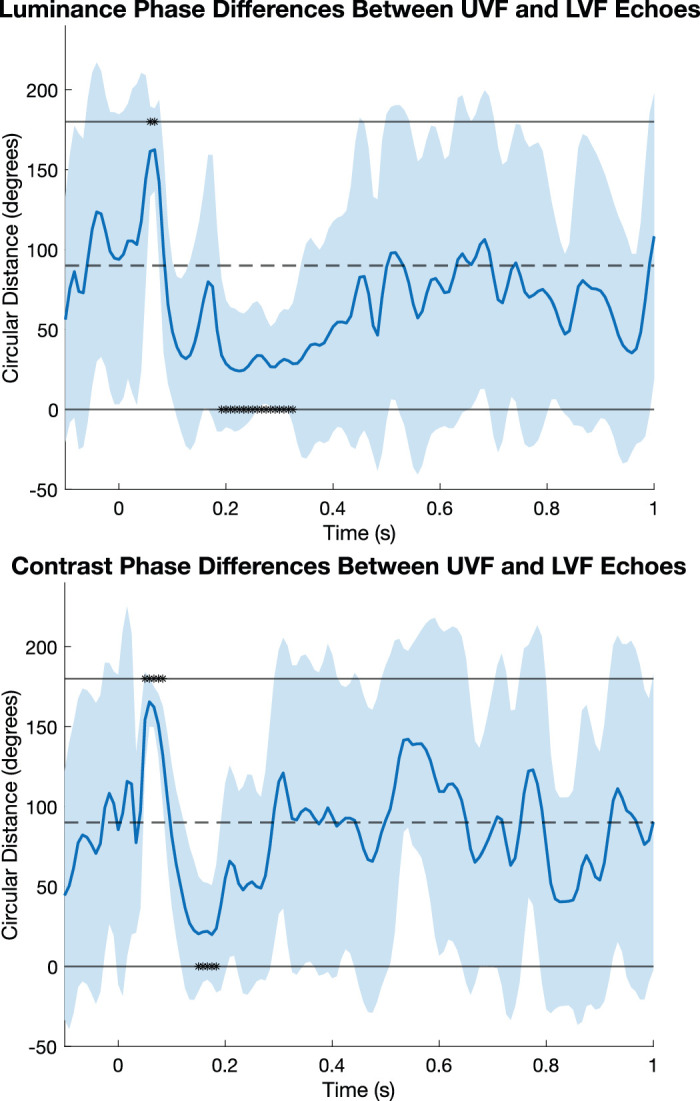
Average phase differences between UVF and LVF echoes for luminance (top) and contrast (bottom), where the circular distance represents whether the UVF and LVF echoes are in phase, meaning there is 0° phase difference (lower solid line in each plot), or whether the echoes are counter-phase, meaning there is 180° phase difference (upper solid line in each plot). Shaded regions indicate bootstrapped 95% confidence intervals.

Phase differences between UVF and LVF echoes were examined to determine whether it was likely that the echoes were staying in V1 or leaving V1 (see Figure 1 for hypothesis). We found phase differences indicating that UVF and LVF echoes were briefly counter-phased at the onset of the luminance echo (Figure 4, top) and the contrast echo (Figure 4, bottom). Specifically, the UVF and LVF luminance echoes were significantly out of phase from 0.042 to 0.096 s (*p*s < 0.05), and the UVF and LVF contrast echoes were significantly out of phase from 0.033 to 0.083 s (*p*s < 0.05). Importantly, this phase difference did not last longer than the expected initial sensory response, and both luminance and contrast UVF and LVF echoes trended more toward being in phase immediately after the period of significant counter-phase activity. In fact, the UVF and LVF luminance echoes become significantly in phase between 0.188 and 0.342 s (*p*s < 0.05), and the UVF and LVF contrast echoes become significantly in phase between 0.125 and 0.192 s (*p*s < 0.05).

The dominant alpha frequencies of upper and lower VF echoes were significantly correlated with each other in the contrast echo (*ρ* = 0.67; *p* = 0.03) but not in the luminance echo (*ρ* = 0.16; *p* = 0.65). Interestingly, the upper and lower VF contrast echoes were both significantly correlated with resting IAF (upper: *ρ* = 0.66, *p* = 0.03; lower: *ρ* = 0.69, *p* = 0.02), but only the UVF luminance echoes were (upper: *ρ* = 0.77, *p* = 0.01; lower: *ρ* = 0.32, *p* = 0.37). The mean alpha frequencies of both echoes were significantly correlated with IAF from the resting data (luminance: *ρ* = 0.79, *p* = 0.01; contrast: *ρ* = 0.69, *p* = 0.02).

## Discussion

This study has replicated and expanded on important findings related to the brain's TRF for low-level visual features. We found a long-lasting alpha-band echo in response to changes in luminance as in earlier work by VanRullen and Macdonald (2012), as well as a shorter-lived alpha-band echo in response to changes in contrast values, where overall luminance was held constant. Both echoes had significant amplitude for at least the first few hundred milliseconds (∼0.3 s), and both echoes were significantly correlated with resting IAF. However, the fact that the luminance echo lasted around 700 ms longer (∼1 s) is a new result. This could be due to the reduced activity in contrast-sensitive channels alone relative to activating luminance and contrast channels in one signal. It is also possible that the complexity of the contrast stimulus we used activated a broader set of neurons with varying orientation preferences, which could have induced more complex dipole patterns and led to some signal cancellation relative to the simple luminance stimulus. While it is unclear the exact mechanism underlying the length of the alpha echoes, future research could explore additional low-level visual features, both in isolation and in combination, to better understand the features that drive these echoes.

Our other critical finding was that both the luminance and contrast echoes displayed significant phase differences between the upper and lower VF stimuli, but only during the initial part of the echo, from around 30–80 ms. This pattern of phase differences followed the expected distribution from activation of the calcarine fissure in V1 (Kelly et al., 2013). This initial phase difference was followed quickly by a significant phase alignment of the upper and lower VF echoes around 100–300 ms. It is likely that these echoes may also occur in higher areas V2 and V3, which also exhibit polarity reversals, but given the similar activation pattern, it is difficult to determine without source localization (Ales et al., 2013). The initial phase difference, at least, suggests that both contrast and luminance echoes originate in V1, but the later phase alignment suggests that the echoes do not remain in V1. The significant phase alignment in the echo suggests the echoes quickly propagated downstream to other visual areas whose electrical activity does not show a UVF/LVF polarity reversal at the scalp. While our approach did not circumvent the possibility that two separate alpha generators were underlying the appearance of a traveling wave, the results provide additional evidence for the notion that alpha echoes begin in primary visual cortex but quickly manifest elsewhere (Lozano-Soldevilla & VanRullen, 2019). Ultimately, our data are consistent with both interpretations, with early occipital sources showing phase inversion and later extrastriate or parietal sources showing in-phase echoes, but they also provide novel evidence for V1 activation in the TRF.

Despite the initial polarity reversals in the upper and lower VF echoes, these echoes tended to have many other similar characteristics. For both luminance and contrast stimuli, the upper and lower VF echoes did not significantly differ in amplitude at any point during the actual echo. Only the contrast echoes briefly differed by visual field, long after the echo had subsided (around 975 ms). Additionally, the upper and lower VF contrast echoes significantly correlated with each other and with alpha frequencies. While a similar pattern held for the luminance echoes, their dominant frequencies were not as strongly correlated across visual fields or with IAF. Overall, these echoes seem to be fairly consistent across visual fields, but their duration depends strongly on the specific stimulus features driving them (i.e., contrast vs. luminance).

It has been suggested that the long-lasting nature of alpha-band echoes could support a form of iconic visual memory. If this is true, our results would predict that iconic memory for contrast-defined targets should decay considerably faster than for luminance (and contrast) defined targets, yet this has not clearly been shown (Adelson & Jonides, 1980). Additionally, we have shown that the alpha echoes originate in V1 and seem to quickly move out of V1, perhaps indicating that the echoes are not solely maintaining visual information but are facilitating the spread of visual information through the visual hierarchy. Prior work showing that alpha echoes are likely traveling from posterior to anterior regions (Lozano-Soldevilla & VanRullen, 2019; Luo, VanRullen, & Alamia, 2021) was also supported by functional magnetic resonance imaging data that indicated the alpha echoes were originating in early visual cortex (Luo, Brüers, Berry, VanRullen, & Reddy, 2021). However, it is unclear how these alpha echoes relate to downstream activity to modulate our perceptual experience or other cognitive factors. Future research should therefore explore how these alpha echoes may interact with downstream processes, with iconic memory and temporal integration being potential candidate functions.
